# Association of Nongenetic Factors With Breast Cancer Risk in Genetically Predisposed Groups of Women in the UK Biobank Cohort

**DOI:** 10.1001/jamanetworkopen.2020.3760

**Published:** 2020-04-24

**Authors:** Kawthar Al Ajmi, Artitaya Lophatananon, Krisztina Mekli, William Ollier, Kenneth R. Muir

**Affiliations:** 1 Division of Population Health, Health Services Research and Primary Care, Faculty of Biology, Medicine and Health, Centre for Epidemiology, University of Manchester, Manchester, United Kingdom; 2 Cathie Marsh Institute for Social Research, School of Social Sciences, Faculty of Humanities, University of Manchester, Manchester, United Kingdom; 3 School of Healthcare Science, Faculty of Science and Engineering, Manchester Metropolitan University, Manchester, United Kingdom

## Abstract

**Importance:**

The association between noninherited factors, including lifestyle factors, and the risk of breast cancer (BC) in women and the association between BC and genetic makeup are only partly characterized. A study using data on current genetic stratification may help in the characterization.

**Objective:**

To examine the association between healthier lifestyle habits and BC risk in genetically predisposed groups.

**Design, Setting, and Participants:**

Data from UK Biobank, a prospective cohort comprising 2728 patients with BC and 88 489 women without BC, were analyzed. The data set used for the analysis was closed on March 31, 2019. The analysis was restricted to postmenopausal white women. Classification of healthy lifestyle was based on Cancer Research UK guidance (healthy weight, regular exercise, no use of hormone replacement therapy for more than 5 years, no oral contraceptive use, and alcohol intake <3 times/wk). Three groups were established: favorable (≥4 healthy factors), intermediate (2-3 healthy factors), and unfavorable (≤1 healthy factor). The genetic contribution was estimated using the polygenic risk scores of 305 preselected single-nucleotide variations. Polygenic risk scores were categorized into 3 tertiles (low, intermediate, and high).

**Main Outcomes and Measures:**

Cox proportional hazards regression was used to assess the hazard ratios (HRs) of the lifestyles and polygenic risk scores associated with a malignant neoplasm of the breast.

**Results:**

Mean (SD) age of the 2728 women with BC was 60.1 (5.5) years, and mean age of the 88 489 women serving as controls was 59.4 (4.9) years. The median follow-up time for the cohort was 10 years (maximum 13 years) (interquartile range, 9.44-10.82 years). Women with BC had a higher body mass index (relative risk [RR], 1.14; 95% CI, 1.05-1.23), performed less exercise (RR, 1.12; 95% CI, 1.01-1.25), used hormonal replacement therapy for longer than 5 years (RR, 1.23; 95% CI, 1.13-1.34), used more oral contraceptives (RR, 1.02; 95% CI, 0.93-1.12), and had greater alcohol intake (RR, 1.11; 95% CI, 1.03-1.19) compared with the controls. Overall, 20 657 women (23.3%) followed a favorable lifestyle, 60 195 women (68.0%) followed an intermediate lifestyle, and 7637 women (8.6%) followed an unfavorable lifestyle. The RR of the highest genetic risk group was 2.55 (95% CI, 2.28-2.84), and the RR of the most unfavorable lifestyle category was 1.44 (95% CI, 1.25-1.65). The association of lifestyle and BC within genetic subgroups showed lower HRs among women following a favorable lifestyle compared with intermediate and unfavorable lifestyles among all of the genetic groups: women with an unfavorable lifestyle had a higher risk of BC in the low genetic group (HR, 1.63; 95% CI, 1.13-2.34), intermediate genetic group (HR, 1.94; 95% CI, 1.46-2.58), and high genetic group (HR, 1.39; 95% CI, 1.11-1.74) compared with the reference group of favorable lifestyle. Intermediate lifestyle was also associated with a higher risk of BC among the low genetic group (HR, 1.40; 95% CI, 1.09-1.80) and the intermediate genetic group (HR, 1.37; 95% CI, 1.12-1.68).

**Conclusions and Relevance:**

In this cohort study of data on women in the UK Biobank, a healthier lifestyle with more exercise, healthy weight, low alcohol intake, no oral contraceptive use, and no or limited hormonal replacement therapy use appeared to be associated with a reduced level of risk for BC, even if the women were at higher genetic risk for BC.

## Key Points

**Question:**

Is adhering to healthier lifestyle habits associated with a reduced breast cancer risk even among genetically predisposed groups?

**Findings:**

This cohort study evaluated 2728 women with breast cancer and 88 489 controls and noted lower risks of breast cancer among women who practice a healthy lifestyle. Factors included in this lifestyle were exercise, healthy weight, low alcohol intake, and no oral contraceptive use, as well as avoiding or limiting use of hormonal replacement therapy to less than 5 years, among low, intermediate, and high genetic risk groups.

**Meaning:**

Following a healthier lifestyle appears to be associated with a decreased level of risk of breast cancer across all strata of genetic risk.

## Teaser

This cohort study examines the association of nongenetic factors, such as lifestyle habits, with risk of breast cancer in women with varying levels of genetic predisposition for developing the disease.

## Introduction

Breast cancer (BC) is the most common cancer in women as well as the second most common cause of cancer-related death in women.^1,2^ In the UK, it is estimated that more than 55 000 new cases of BC occur annually.^2^ Both genetic and lifestyle factors play crucial roles in the complex mechanism of BC. Evidence supporting the genetic component of BC is seen with highly penetrant rare gene variants, such as in the *BRCA1* and *BRCA2* genes. These particular variants, however, account for just a small proportion (<5%) of BC cases^3^ and for 1.5% to 2% in familial BC cases.^4^ Genome-wide association studies have identified a number of single-nucleotide variations (SNVs) associated with risk for BC development, although these SNVs individually contribute only a small genetic proportion or are in genes exhibiting medium to low penetrance. The cumulative genetic contribution and effects of all such BC-associated SNVs is referred to as a polygenic risk score (PRS). This aggregated PRS is present in a substantial proportion of all patients with BC (88%).^5,6,7^ The application of genetic risk stratification to individuals as a clinical tool for aiding BC screening is now on the horizon.^6^ Mavaddat et al^8^ showed that women at the top 5% of the PRS can develop BC at age 37 years, while those in the lowest 20% of the PRS will likely never develop BC.

Some lifestyle and behavioral factors can play an important role in and contribute to the risk of BC.^9,10,11,12,13,14^ However, few studies have investigated the contribution and role of lifestyle risk exposures in BC in women exhibiting different PRSs. Whereas inherited genetic risk for disease is not modifiable, this factor is not the case for most known nongenetic risk factors. The central hypothesis examined in this study is that, regardless of a person’s PRS, overall BC risk can be reduced by following a favorable lifestyle.

## Methods

Data from women within the UK Biobank longitudinal cohort study were used. The data set for the analysis was closed on March 31, 2019. The UK Biobank is a national cohort including 502 650 men and women aged between 39 and 71 years. Patients were enrolled between 2006 and 2010 and continue to be longitudinally followed up for capture of subsequent health events. Participants gave the UK Biobank written informed consent to use their data and samples for health-related research purposes. Ethics approval for use of UK Biobank data was obtained from the North West-Haydock research ethics committee. This study followed the Strengthening the Reporting of Observational Studies in Epidemiology (STROBE) reporting guideline for cohort studies.

In this analysis, the inclusion criteria to select study participants were (1) British women who were white (age, 40-71 years), (2) postmenopausal women who did not report a history of hysterectomy or bilateral oophorectomy and reported no longer menstruating, and (3) women with a menopause age of 40 years or older. Deceased participants were excluded from our analysis. Of the UK Biobank cohort of 273 402 female participants, 114 723 women (42.0%) fulfilled our inclusion criteria.

The study outcome was defined as women with a malignant neoplasm of the breast. Cases and controls were identified according to the criteria summarized in eFigure 1 in the Supplement. We used 3 coding systems to identify patients with BC and those serving as controls: *International Statistical Classification of Diseases and Related Health Problems, Tenth Revision*; *International Classification of Diseases, Ninth Revision*; and self-reported (eTable 1 in the Supplement). If patients with breast cancer appeared to have an incident case of BC according to any of these 3 coding systems, they were deemed incident cases (age at cancer diagnosis was older than age when they attended the assessment center of the UK Biobank study). Cases were considered prevalent only if they were defined as such according to any of the 3 coding systems, which was applicable only if none of the 3 approaches had described the BC case as being an incident case. A total of 2728 postmenopausal women with incident cases of BC were eligible for the analysis. Controls were defined as women without a history of any cancer, carcinoma in situ, or unknown neoplasm. The final number of controls selected by menopausal status and our set criteria was 88 489. eFigure 1 in the Supplement illustrates the number of study participants in the case and control selection process.

Cancer Research UK^15^ has reported risk factors for BC development as being either modifiable or nonmodifiable. Based on their list, we identified the 5 modifiable factors: weight, alcohol intake, physical activity, oral contraceptive use, and hormonal replacement therapy (HRT) intake for more than 5 years. We developed a scoring system based on the presence or absence of these 5 factors to derive favorable lifestyle, intermediate lifestyle, and unfavorable lifestyle. This approach was adopted from similar studies on coronary heart disease^16^ and dementia.^17^ The details of the 5 factors and score definition are presented in Table 1. Eligible participants were stratified into 3 categories: favorable lifestyle (≥4 healthy factors present), intermediate lifestyle (2 or 3 healthy factors present), and unfavorable lifestyle (≤1 healthy factor present).

**Table 1.** Criteria for Healthy Lifestyle Classification

Factor	UK Biobank cohort	Code
**Healthy lifestyle criteria**		
Healthy weight	Healthy: BMI <25	1
	Unhealthy: BMI ≥25	0
Regular physical activity	Healthy: ≥1 time/wk	1
	Unhealthy: none	0
Alcohol intake	Healthy: none or <3 times/wk	1
	Unhealthy: ≥3 times/wk	0
Oral contraceptive use	Healthy: none	1
	Unhealthy: any use	0
Hormone replacement therapy	Healthy: none or <5 y	1
	Unhealthy: use for ≥5 y	0
**Lifestyle classification**		
Favorable	Presence of 4-5 healthy lifestyle factors	Sum: ≥4
Intermediate	Presence of 2-3 healthy lifestyle factors	Sum: 2 or 3
Unfavorable	Presence of ≤1 healthy lifestyle factor	Sum: 0 or 1

Abbreviations: BMI, body mass index (calculated as weight in kilograms divided by height in meters squared).

A PRS was derived based on the Mavaddat score^5^ using the UK Biobank high-density genome-wide SNV data set available for 488 377 of their participants. The SNV data were used from individuals who were included on the basis of being female (matched genetic and self-reported sex) and their genetic ethnic grouping (white). During the quality control process, individuals with missingness (>2%), outliers for heterozygosity, and duplicates, as well as those who were biologically related, were excluded.

The PRS for BC was constructed using the 313 SNVs previously determined to contribute some risk by the hard threshold approach used by Mavaddat et al.^5^ Of these 313 SNVs, 306 were present in the UK Biobank data set; however, SNV rs10764337 was triallelic and excluded. The final number of SNVs used for PRS construction was therefore 305, and their details are presented in eTable 2 in the Supplement. Forty of 305 SNVs had been directly genotyped and successfully passed the marker test applied by UK Biobank.^18^ The remaining 265 SNVs had been imputed. The quality of the imputation was estimated using the information scores available, which is a number between 0 and 1 where 0 indicates complete uncertainty and 1 indicates complete certainty. The lowest information score was 0.86. Linkage disequilibrium was assessed, and no *r*^2^ value between any 2 SNVs reached 0.9. Plinkopen source software version 1.90 was used to carry out the quality control processes.^19^

Individual participant PRS was created by adding the number of risk alleles at each SNV and then multiplying the sum by the effect size as the previously published estimated effect size.^5^ The raw PRS was standardized by dividing each raw PRS by the SD of the PRS derived from the control group. No transformation to the PRS data was applied because the scores were normally distributed (eFigure 2 in the Supplement). A tertile genetic risk classification using standardized PRS values from controls was generated. Each participant was then assigned to a genetic risk group: low (1st tertile up to 33.33%), intermediate (2nd tertile between 33.34% and 66.67%), and high (3rd tertile from 66.68% to 100%).

### Statistical Analysis

Relative risks (RRs) and 95% CIs of the basic risk factors were computed with an adjustment for age and family history using a binomial generalized linear regression model. Cox proportional hazards regression was applied to assess the hazard ratios (HRs) of the lifestyles and BC risk. We first computed HRs for each genetic stratum with the low genetic risk group as a reference group and for each lifestyle (favorable, intermediate, and unfavorable) stratum with the favorable category as a reference group. The HRs in each lifestyle stratum were calculated within each genetic risk group. All analyses were adjusted for age and family history. The Cox proportional hazards regression model assumption for each analysis was tested. A 2-sided *P* value <.05 was considered significant. The Ltable^20^ command was used to compute a 10-year cumulative BC incidence for each lifestyle category within each genetic risk stratum. Results presented in graphic bar charts were generated using Microsoft Excel 2016 (Microsoft Corp).^21^ All analyses were performed using Stata/MP software version 14 (StataCorp LLC).^22^

## Results

The median follow-up time for the cohort was 10 years (maximum, 13 years) (interquartile range, 9.44-10.82 years). The total number of the incident cases was 2728 patients with BC, and the total number of controls was 88 489. The mean (SD) age of the patients was 60.1 (5.5) years and for controls was 59.4 (4.9) years. The mean (SD) body mass index (BMI) measures (calculated as weight in kilograms divided by height in meters squared) were 27.3 (5.0) for patients and 26.9 (4.9) for controls. In addition, patients used more HRT (30.4%) compared with controls (25.2%). Furthermore, women with BC more often reported no regular physical activity (13.3%) compared with controls (12.0%).

Table 2 presents the distribution of the general characteristics and estimated RR results. A 1-year increase in age was associated with a 2.3% increase in BC development risk. Having 1 female first-degree family member (either mother or sister) with BC was associated with a 48.6% increase in BC risk, while having both mother and sister affected was associated with a doubling of the risk of BC compared with women without a family history of BC. An unhealthy weight (BMI ≥25) was associated with a 13.9% increased risk of BC (RR, 1.14; 95% CI, 1.05-1.23). Participants who reported that they did not have regular physical activity were had a 12.2% increased risk of BC (RR, 1.12; 95% CI, 1.01-1.25), and alcohol intake 3 or more times per week was associated with an increased BC risk of 10.7% (RR, 1.11; 95% CI, 1.03-1.19). Use of HRT for 5 or more years was associated with an increased BC risk of 22.9% (RR, 1.23; 95% CI, 1.13-1.34). History of oral contraceptive use did not show any association with BC risk among women in the UK Biobank (RR, 1.02; 95% CI, 0.93-1.12); however, this factor was retained as part of lifestyle classification. Overall, 20 657 women (23.3%) followed a favorable lifestyle, 60 195 women (68.0%) followed an intermediate lifestyle, and 7637 women (8.6%) followed an unfavorable lifestyle. Intermediate and unfavorable lifestyles were both associated with higher risk of BC compared with the favorable lifestyle (intermediate: RR, 1.25; 95% CI, 1.13-1.37; unfavorable: RR, 1.44; 95% CI, 1.25-1.65).

**Table 2.** Relative Risks for Basic Characteristics, Lifestyles, and Genetic Categories

Risk factor	Frequency, No. (%)		RR (95% CI)
	Cases	Controls	
Age^a^	2728 (2.99)	88 489 (97.01)	1.02 (1.02-1.03)
Family history^b^			
No family history	2276 (83.80)	78 408 (88.84)	1 [Reference]
Mother or sister BC history	412 (15.17)	9405 (10.66)	1.49 (1.34-1.65)
Mother and sister BC history	28 (1.03)	440 (0.50)	2.10 (1.46-3.01)
Weight			
Healthy	995 (36.55)	35 537 (40.25)	1 [Reference]
Unhealthy	1727 (63.45)	52 749 (59.75)	1.14 (1.05-1.23)
Regular physical activity			
≥1 time/wk	2329 (86.74)	76 466 (88.00)	1 [Reference]
No physical activity	356 (13.26)	10 423 (12.00)	1.12 (1.01-1.25)
Alcohol intake			
No intake or <3 times/wk	1566 (57.40)	52 892 (59.80)	1 [Reference]
Intake ≥3 times/wk	1162 (42.60)	35 557 (40.20)	1.11 (1.03-1.19)
Oral contraceptive intake			
No	561 (20.58)	17 240 (19.50)	1 [Reference]
Yes	2165 (79.42)	71 149 (80.50)	1.02 (0.93-1.12)
HRT intake			
No	1895 (69.64)	66 093 (74.82)	1 [Reference]
Yes	826 (30.36)	22 244 (25.18)	1.23 (1.13-1.34)
Healthy lifestyle score			
Favorable	530 (19.43)	20 657 (23.34)	1 [Reference]
Intermediate	1909 (69.98)	60 195 (68.03)	1.25 (1.13-1.37)
Unfavorable	289 (10.59)	7637 (8.63)	1.44 (1.25-1.65)
PRS category			
Low	440 (19.67)	24 297 (33.70)	1 [Reference]
Intermediate	655 (29.28)	23 983 (33.27)	1.49 (1.32-1.68)
High	1142 (51.05)	23 814 (33.03)	2.55 (2.28-2.84)

Abbreviations: BC, breast cancer; HRT, hormone replacement therapy; PRS, polygenic risk score; RR, relative risk.

^a^ No adjustment.

^b^ Adjusted for age only.

The mean standardized PRS of the cases was 26.26 (range, 21.63-29.40), which is higher than the mean standardized PRS of the control group (25.807; range, 21.119-29.941). This difference was examined using a *t* test, and a significant difference between the mean score was apparent between cases and controls (*P* < .001). Moreover, the estimated HR for overall BC among postmenopausal women per unit of increased PRS was 1.55 (95% CI, 1.48-1.61). Analysis of the PRS tertile groups indicated a gradient of increased BC risk across tertiles (for second tertile vs first tertile, *P* < .001; for third tertile vs first tertile, *P* < .001). Women in the higher genetic risk group (3rd tertile) were at significantly higher risk of BC (RR, 2.55; 95% CI, 2.28-2.84) compared with women in the low genetic risk group after adjusting for age and family history. Similarly, women in the intermediate risk group showed a moderate increased risk (RR, 1.49; 95% CI, 1.32-1.68) compared with those in the low genetic risk group.

**Figure 1.**
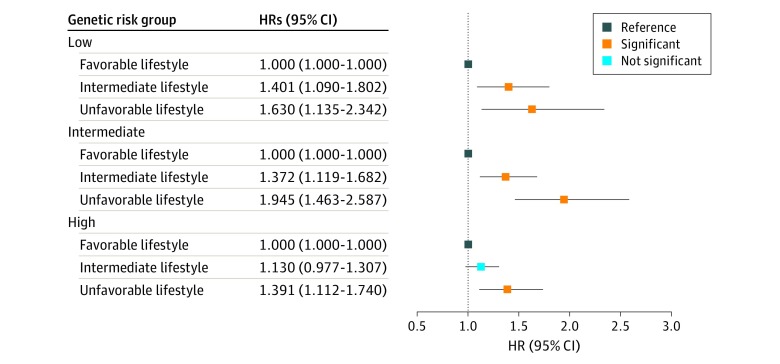
Association of Breast Cancer With Lifestyle and Genetic Factors The hazard ratio (HR) of each genetic group was stratified based on the favorable, intermediate, and unfavorable lifestyles, with favorable lifestyle as the reference group in the 3 genetic groups.

Results of estimated HRs for lifestyle and BC risk in each genetic risk group are presented in Table 3. The results of Cox proportional hazards regression model assumption testing in the low, intermediate, and high genetic risk groups suggested no statistically significant violation of Cox proportional hazards regression model assumption. In the low genetic risk group, significantly increased HRs were observed in both the unfavorable lifestyle (HR, 1.63; 95% CI, 1.13-2.34) and intermediate lifestyle (HR, 1.40; 95% CI, 1.09-1.80) groups compared with the favorable lifestyle group. In the intermediate genetic risk group, significantly increased HRs were shown in the unfavorable (HR, 1.94; 95% CI, 1.46-2.58) and intermediate (HR, 1.37; 95% CI, 1.12-1.68) lifestyle groups. In the higher genetic risk strata, a significant HR was observed in the unfavorable lifestyle group (HR, 1.39; 95% CI, 1.11-1.74) compared with favorable lifestyle. All of the above results suggest that, within the same genetic risk group, adhering to a less healthy lifestyle (intermediate and unfavorable lifestyle) is associated with an increased risk of BC. Figure 1 shows a forest plot of HRs according to genetic risk group and lifestyle categories.

**Table 3.** Breast Cancer HRs Based on Lifestyles, Stratified by the Genetic Risk Group

Genetic risk group	Healthy lifestyle score^a^	Frequency, No. (%)		HR (95% CI)
		Cases	Controls	
Low	Favorable lifestyle	75 (17.05)	5550 (22.84)	1 [Reference]
	Intermediate lifestyle	317 (72.05)	16 540 (68.07)	1.40 (1.09-1.80)
	Unfavorable lifestyle	48 (10.91)	2204 (9.08)	1.63 (1.14-2.34)
	PH assumption *P* value	.99		
	*P* value	.004		
Intermediate	Favorable lifestyle	117 (17.86)	5582 (23.27)	1 [Reference]
	Intermediate lifestyle	458 (69.92)	16 336 (68.11)	1.37 (1.12-1.68)
	Unfavorable lifestyle	80 (12.21)	2065 (8.61)	1.94 (1.46-2.58)
	PH assumption *P* value	.08		
	*P* value	<.001		
High	Favorable lifestyle	236 (20.67)	5571 (23.39)	1 [Reference]
	Intermediate lifestyle	792 (69.35)	16 278 (68.35)	1.13 (0.98-1.31)
	Unfavorable lifestyle	114 (9.98)	1965 (8.25)	1.39 (1.11-1.74)
	PH assumption *P* value	.69		
	*P* value	.007		

Abbreviations: BC, breast cancer; HR, hazard ratio; PH, proportional hazards.

^a^ Adjusted for age and family history of BC.

**Figure 2.**
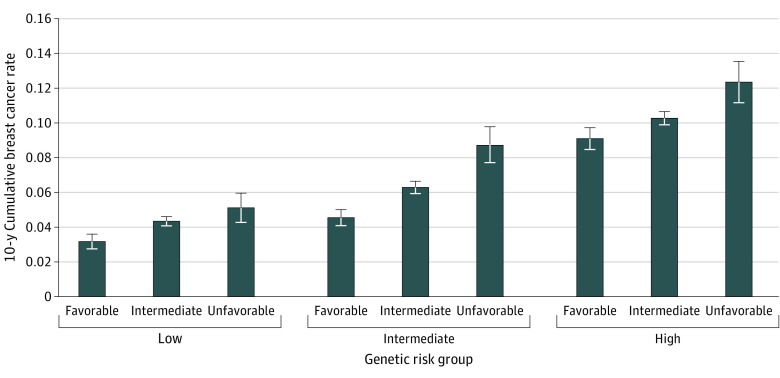
Ten Year Cumulative Breast Cancer Incidence Rate of UK Biobank Postmenopausal Women, Classified According to Genetic and Lifestyle Factors The error bars represent the mean rate with the maximum and minimum incidence rate.

The results of the 10-year cumulative incidence rate of BC in all genetic risk groups suggest incremental rates of increase from favorable to intermediate to unfavorable (Figure 2) lifestyle. A favorable lifestyle had the lowest 10-year cumulative BC incidence rate across all genetic risk groups (low, 3%; intermediate, 5%; and high, 9%). Similar findings in the 10-year cumulative BC incidence rate were observed for an unfavorable lifestyle across the genetic risk groups (low, 5%; intermediate, 9%; and high, 12%).

## Discussion

It has been estimated that BC could be prevented in 23% of patients in the UK.^2^ Thus, it is important to understand the contribution of modifiable risk factors to BC and how they affect or add to the inherited genetic factors. This study therefore investigated the association between genetic and lifestyle factors with BC risk and tested the hypothesis that BC risk in postmenopausal women can be modified or reduced by improving lifestyle habits, even for the highest genetic risk group. We opted to investigate our hypothesis only in postmenopausal women because of the high proportion of BC incidence and prevalence in this group.^15,23^ Furthermore, BC in premenopausal women is usually a more aggressive disease, likely caused by high penetrance genes,^24,25,26,27^ resulting in a less-favorable prognosis.^28^

This study used genetic and lifestyle data generated by UK Biobank, a longitudinal study of the contribution of genetic, environmental, and lifestyle risk factors in disease. Participants were grouped by their level of polygenetic risk for BC using the SNV data available within the UK Biobank database. The 305 SNVs included in the PRS were mainly common variants with limited contribution to BC risk. Aggregated effect sizes of these SNVs were used to develop a standardized PRS.

Although many risk/protective factors contribute to BC development,^29^ we selected 5 robust modifiable risk factors, recognized previously by Cancer Research UK as being associated with BC in white females.^9,30,31,32^ The frequencies of these modifiable risk factors are high in women in the UK, and if they can be modified can potentially reduce BC incidence. The prevalence of these 5 modifiable risk factors in the UK Biobank female cohort were as follows: 63.4% exhibiting unhealthy weight in patients with BC vs 59.8% in controls, 13.3% of patients with BC having no regular exercise vs 12.0% of controls, 42.6% of patients with BC with regular alcohol intake vs 40.2% of controls, 79.4% of patients with BC who used oral contraceptives vs 80.5% of controls, and 30.4% of patients with BC who received HRT vs 25.2% of controls.

The findings from other large cohorts, including the Million Women Study and the Breast Cancer Association Consortium, have indicated that BC risk increase is associated with unhealthy weight,^9,12,33^ no or limited exercise,^12,13^ level of alcohol intake,^12,13,14^ use of oral contraceptives,^9,12^ and use of HRT.^9,10,11,12,34^ The Cancer Research UK suggested that the relative contributions of these factors to BC development are 2% for HRT, 8% for obesity, 8% for alcohol intake, and less than 1% for use of oral contraceptives.^2^ The results from our study are in keeping with the Cancer Research UK in that maintaining a healthy weight is associated with reduced BC risk by 13.9%, participating in regular exercise is associated with reduced BC risk by 12.2%, maintaining alcohol intake at less than 3 times a week is associated with reduced BC risk by 10.7%, and avoiding HRT use is associated with reduced BC risk by 22.9%. Our findings therefore support the selection of these modifiable lifestyle risk factors for BC, with the exception of oral contraceptive use. Thus, further studies are needed to investigate whether there is a causal association between new risk factors and BC using, for example, a mendelian randomization approach.

Even though oral contraceptive use has been suggested previously to be associated with BC, this risk factor did not show any association in our study. Possible explanations for this observation could be that we did not take into account other related factors that could be associated with the results, including the type of oral contraceptive used,^35^ the duration of use,^36^ and age at the time when the drugs were stopped.^37^ Furthermore, women who have had human chorionic gonadotropin injections as part of infertility or weight loss treatments showed a lower risk of BC.^38^ All of these factors may have implications in BC risk. For example, if women stopped oral contraceptive use for more than 10 years before their enrollment in the UK Biobank study, their BC risk will be reduced or returned to the same risk of women who never used oral contraceptives.^37^

Exhibiting 2 or 3 of these healthy lifestyle factors (intermediate lifestyle) was associated with increased risk of BC by 24.5% compared with an increase of 43.6% in women who adhered to none or 1 of these factors (unfavorable lifestyle). Our findings suggest that women may be able to alter or reduce their risk of developing BC by following healthier lifestyles. While we did not set out to look for a formal interaction owing to limited study power, the results showed no significant interaction between lifestyles and genetic risk groups, and the 2 variables were considered as independent in the analysis. Further analysis demonstrated that a high PRS was associated with higher risk of BC. This level of increased risk is in line with other published findings.^5^ The HRs derived from our analysis were generated by including only postmenopausal women. In contrast, the study by Mavaddat et al^5^ reported HRs that were derived from both premenopausal and postmenopausal women.

The beneficial risk-reducing association of adhering to healthy lifestyles across all genetic risk stratification groups supports our hypothesis that BC risk reduction is seen regardless of the effect size of the PRS. We also found an association between 10-year cumulative BC incidence rate and both lifestyle and genetic factors when assessed together. This increase suggests that BC incidence may be reduced by following favorable lifestyles even in women with high genetic risk.

This study suggests that the lifestyle followed by women may contribute to reducing the incidence of BC in those who have an increased genetic predisposition for this condition. Similar approaches have been used to investigate complex risk factors associated with dementia^17^ and coronary heart disease.^16^ Both studies came to a conclusion similar to ours. In the dementia study, by adhering to favorable lifestyles (no current smoking, moderate alcohol intake, healthy diet, and regular exercise), the level of dementia was reduced. Similarly, in coronary artery disease, no smoking, no obesity, healthy diet, and regular exercise were associated with a reduction in the extent of coronary heart disease in participants, and this result was also observed in patients within the highest PRS group.

### Strengths and Limitations

A strength of our study is that a large sample size was analyzed and the selection of participants was spread across the UK. Furthermore, the quality and comprehensive nature of the phenotypic exposures assessed by UK Biobank were robust and of high standards. Our use of a prospective study design allowed exposure assessment before BC development in the cohort. However, the study has some limitations. The PRS used was restricted to white women and therefore presents a limitation on its generalizability to a wider range of racial/ethnic groups. Additional validation of these PRSs in other populations is needed to further understand its utility in genetic risk stratification. Our analysis was restricted to postmenopausal women; therefore, these results cannot be applied to premenopausal women.

However, the benefits reported herein for healthy lifestyle factors may also be seen in younger women. In addition, our analysis did not investigate the various known pathologic-based subtypes of BC, including *ER* positive and negative, *PR* positive and negative, and *ERBB2* (formerly *HER2* or *Her2/neu*) positive and negative.

## Conclusions

The results of this study suggest that promotion of healthy lifestyles through adequate levels of exercise, healthy weight, no or limited alcohol intake, and avoidance of hormonal replacement therapy should be encouraged to reduce the risk of BC. Following a healthy lifestyle appears to be associated with a reduced level of BC risk in all 3 genetic risk strata, further illustrating the importance of lifestyle factors in common diseases with a genetic predisposition, such as BC.

## Notes

Supplement. **eFigure 1.** Number of Participants in Each Filter Step **eFigure 2.** Distribution of Polygenic Risk Scores **eTable 1.** Identification Codes for Patients With Breast Cancer and Controls in UK Biobank Cohort **eTable 2.** 305 SNPs Used in Calculating the Polygenic Risk Scores of the UK Biobank Females
