# Supplementary material for: Association of Nongenetic Factors With Breast Cancer Risk in Genetically Predisposed Groups of Women in the UK Biobank Cohort
Source: JAMA Netw Open. 2020 Apr 24;3(4):e203760. doi: 10.1001/jamanetworkopen.2020.3760 (PMC7182796; doi:10.1001/jamanetworkopen.2020.3760)
Supplement: Supplement. — eFigure 1. Number of Participants in Each Filter Step eFigure 2. Distribution of Polygenic Risk Scores eTable 1. Identification Codes for Patients With Breast Cancer and Controls in UK Biobank Cohort eTable 2. 305 SNPs Used in Calculating the Polygenic Risk Scores of the UK Biobank Females [file jamanetwopen-3-e203760-s001.pdf]

Supplementary Online Content

Al Ajmi K, Lophatananon A, Mekli K, Ollier W, Muir KR. Association of nongenetic factors with breast cancer risk in genetically predisposed groups of women in the UK Biobank cohort. *JAMA Netw Open*. 2020;3(4):e203760. doi:10.1001/jamanetworkopen.2020.3760

eFigure 1. Number of Participants in Each Filter Step

eFigure 2. Distribution of Polygenic Risk Scores

eTable 1. Identification Codes for Patients With Breast Cancer and Controls in UK Biobank Cohort

eTable 2. 305 SNPs Used in Calculating the Polygenic Risk Scores of the UK Biobank Females

This supplementary material has been provided by the authors to give readers additional information about their work.

© 2020 Al Ajmi K et al. *JAMA Network Open.*

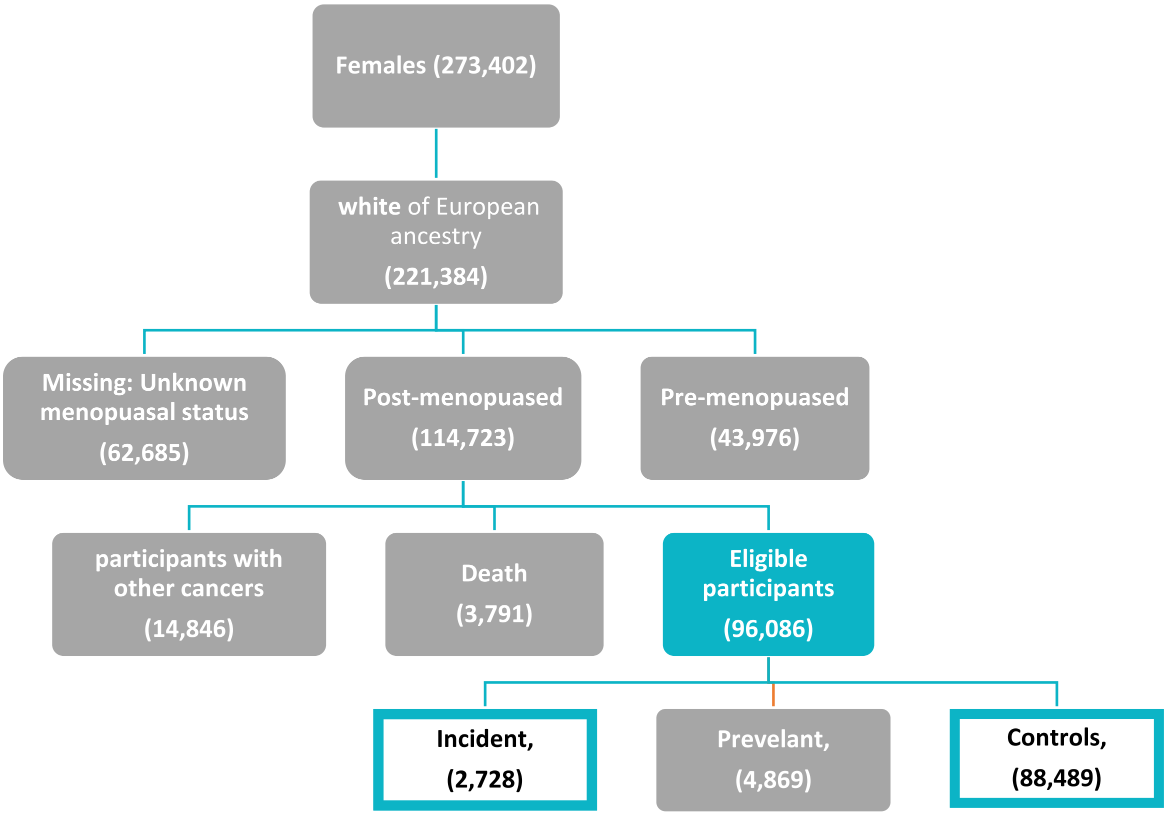

eFigure 1. Number of participants in each filter step.

© 2020 Al Ajmi K et al. *JAMA Network Open.*

eFigure 2. Distribution of Polygenic Risk Scores

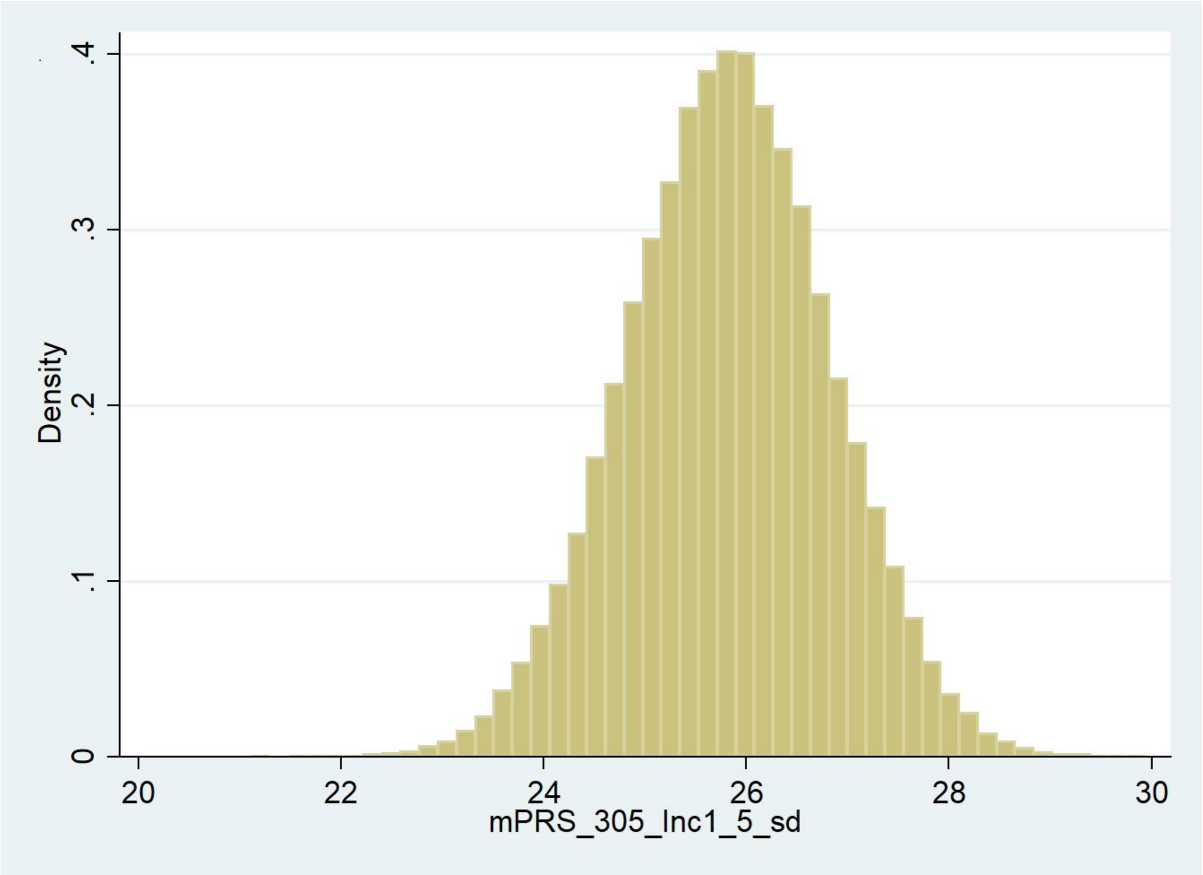

© 2020 Al Ajmi K et al. *JAMA Network Open.*

eTable 1. Identification codes for patients with breast cancer and controls in UK biobank cohort

Sources	Breast cancer cases		Controls	Participants excluded from the control group	
	Incident	Prevalent	Controls used in the analysis	Participants with cancer history	
				Alive participants with cancer	Death registry
ICD10	Codes start with C50 and its subclasses , C501, C502, C503, C504, C505, C506, C507, C508, and C509		Participants without any other cancer codes from the three sources	Participants with any cancer code (Other than breast cancer) from the three sources either (breast in situ, other in situ, other cancers, and neoplasm of unknown nature or behavior	All dead females were excluded from the analysis regardless of the cause
ICD9	Codes start with 174 and its subclasses 1741, 1742, 1743, 1744, 1745, 1746, 1747, 1748, and 1749				
Self-reported cancers	1002				
Frequency (%)	2,728 (2.38%)	4,869 (3.68%)	88,489 (77.13%)	14,846 (12.94%)	3,791 (3.30)

© 2020 Al Ajmi K et al. *JAMA Network Open.*

eTable 2. 305 SNPs used in calculating the polygenic risk scores of the UK biobank females

eTable 2. 305 SNPs used in calculating the polygenic risk scores of the UK biobank females

CHR	rs_id	BP	A1	A2	MAF	QC-test	Info_score	phase3_1kg_id	rs_number	reference_allele	effect_allele	eaf	overall_breast_cancer
1	rs707475	7917076	A	G	0.395		0.998	rs707475:7917076:G:A	rs707475	G	A	0.390	-0.041
1	rs616488	10566215	G	A	0.345	106		rs616488:10566215:A:G	rs616488	A	G	0.329	-0.059
1	rs2992756	18807339	C	T	0.500		0.999	rs2992756:18807339:T:C	rs2992756	T	C	0.515	-0.056
1	rs4233486	41380440	C	T	0.340		0.986	rs4233486:41380440:C:T	rs4233486	C	T	0.644	0.043
1	rs114282204	41389220	C	T	0.016		0.962	rs114282204:41389220:T:C	rs114282204	T	C	0.017	0.155
1	1:46670206_TC_T	46670206	T	TC	0.302		0.988	rs144105764:46670206:TC:T	rs144105764	TC	T	0.297	0.045
1	1:51467096_CT_C	51467096	C	CT	0.495		0.905	rs56168262:51467096:CT:C	rs56168262	CT	C	0.480	0.037
1	rs17426269	88156923	A	G	0.151		0.980	rs17426269:88156923:G:A	rs17426269	G	A	0.149	0.049
1	rs2151842	88428199	A	C	0.240		0.999	rs2151842:88428199:C:A	rs2151842	C	A	0.248	-0.039
1	rs612683	100880328	T	A	0.401		0.984	rs612683:100880328:A:T	rs612683	A	T	0.410	0.037
1	1:110198129_CAAA_C	110198129	CAAA	C	0.206		0.950	rs56097627:110198129:CAAA:C	rs56097627	CAAA	C	0.776	0.046
1	rs7513707	114445880	A	G	0.174		0.999	rs7513707:114445880:G:A	rs7513707	G	A	0.166	0.062
1	rs12406858	118141492	C	A	0.252		0.989	rs12406858:118141492:A:C	rs12406858	A	C	0.266	0.045
1	rs637868	120257110	T	C	0.471		0.995	rs637868:120257110:T:C	rs637868	T	C	0.531	0.039
1	rs11249433	121280613	G	A	0.417	106		rs11249433:121280613:A:G	rs11249433	A	G	0.405	0.088
1	rs111458676	121287994	G	A	0.087		0.971	rs111458676:121287994:A:G	rs111458676	A	G	0.106	-0.067
1	rs143384623	145604302	CT	C	0.346		0.963	rs72127681:145604302:C:CT	rs143384623	C	CT	0.352	-0.040
1	rs11205303	149906413	C	T	0.410	106		rs11205303:149906413:T:C	rs11205303	T	C	0.402	0.055
1	rs12091730	155556971	A	G	0.232		0.996	rs12091730:155556971:G:A	rs12091730	G	A	0.230	0.050
1	rs761575824	168171052	C	CA	0.086		0.927	rs139315904:168171052:CA:C	rs139315904	CA	C	0.110	-0.068
1	rs11463354	172328767	TA	T	0.311		0.953	rs11463354:172328767:T:TA	rs11463354	T	TA	0.331	-0.044
1	rs35383942	201437832	T	C	0.063	106		rs35383942:201437832:C:T	rs35383942	C	T	0.056	0.092
1	rs6686987	202184600	T	C	0.403		0.995	rs6686987:202184600:C:T	rs6686987	C	T	0.399	-0.007
1	rs7514172	203770448	A	T	0.277		1.000	rs7514172:203770448:T:A	rs7514172	T	A	0.272	0.050

© 2020 Al Ajmi K et al. *JAMA Network Open.*

eTable 2. 305 SNPs used in calculating the polygenic risk scores of the UK biobank females													
CHR	rs_id	BP	A1	A2	MAF	QC-test	Info_score	phase3_1kg_id	rs_number	reference_allele	effect_allele	eaf	overall_breast_cancer
1	rs11268668	204502514	T	TTCTGAAACAGGG	0.214		0.979	rs11268668:204502514:T:TTCTGAAACAGGG	rs11268668	T	TTCTGAAACAGGG	0.803	-0.032
1	rs2785646	208076291	A	G	0.350		0.994	rs2785646:208076291:G:A	rs2785646	G	A	0.334	-0.037
1	rs2576261	217053815	G	T	0.326		0.981	rs2576261:217053815:T:G	rs2576261	T	G	0.328	0.042
1	rs11117758	217220574	A	G	0.210		0.985	rs11117758:217220574:G:A	rs11117758	G	A	0.211	-0.044
1	rs11118563	220671050	T	C	0.229		0.980	rs11118563:220671050:C:T	rs11118563	C	T	0.242	0.042
1	rs72755295	242034263	G	A	0.033		0.985	rs72755295:242034263:A:G	rs72755295	A	G	0.031	0.143
2	rs78425380	10138983	C	T	0.100		0.940	rs78425380:10138983:T:C	rs78425380	T	C	0.116	0.060
2	rs6743383	19315675	T	A	0.440		0.997	rs6743383:19315675:T:A	rs6743383	T	A	0.560	-0.033
2	rs6725517	25129473	G	A	0.431		0.999	rs6725517:25129473:A:G	rs6725517	A	G	0.408	-0.043
2	rs12472404	29179452	C	G	0.232		0.995	rs12472404:29179452:G:C	rs12472404	G	C	0.229	-0.007
2	rs4322799	29615233	C	T	0.231		0.971	rs4322799:29615233:T:C	rs4322799	T	C	0.262	-0.043
2	rs553796823	39699510	CT	C	0.452		0.938	rs11406722:39699510:C:CT	rs11406722	C	CT	0.466	-0.040
2	rs6756513	70172587	A	G	0.283		0.990	rs6756513:70172587:G:A	rs6756513	G	A	0.279	-0.041
2	rs1036759	88358825	C	G	0.301		0.972	rs1036759:88358825:G:C	rs1036759	G	C	0.308	0.047
2	rs6746250	121058254	A	G	0.312		0.998	rs6746250:121058254:A:G	rs6746250	A	G	0.705	-0.033
2	rs17625845	121089731	C	T	0.195	106		rs17625845:121089731:T:C	rs17625845	T	C	0.194	-0.043
2	rs10164550	121159205	A	G	0.354		0.968	rs10164550:121159205:G:A	rs10164550	G	A	0.353	-0.044
2	rs10179592	121246568	T	C	0.098		0.998	rs10179592:121246568:T:C	rs10179592	T	C	0.897	0.099
2	rs17726078	172974566	G	C	0.479	106		rs17726078:172974566:C:G	rs17726078	C	G	0.474	-0.047
2	rs1550622	174212910	A	G	0.167		0.984	rs1550622:174212910:A:G	rs1550622	A	G	0.845	0.059
2	rs2356656	192381934	C	T	0.126		0.995	rs2356656:192381934:C:T	rs2356656	C	T	0.859	0.032
2	rs10197246	202204741	T	C	0.266		0.997	rs10197246:202204741:T:C	rs10197246	T	C	0.721	-0.049
2	rs4442975	217920769	G	T	0.488		0.989	rs4442975:217920769:G:T	rs4442975	G	T	0.500	-0.132
2	2:217955896_GA_G	217955896	G	GA	0.035		0.980	2:217955896:GA:G	rs572022984	GA	G	0.038	-0.202
2	rs11693806	218292158	C	G	0.265		0.994	rs11693806:218292158:C:G	rs11693806	C	G	0.731	-0.076
2	rs3791977	218714845	A	G	0.399		0.955	rs3791977:218714845:G:A	rs3791977	G	A	0.392	-0.043
2	rs4676356	241388857	C	A	0.028	106		rs4676356:241388857:C:A	rs4676356	C	A	0.977	-0.123

© 2020 Al Ajmi K et al. *JAMA Network Open.*

eTable 2. 305 SNPs used in calculating the polygenic risk scores of the UK biobank females

CHR	rs_id	BP	A1	A2	MAF	QC-test	Info_score	phase3_1kg_id	rs_number	reference_allele	effect_allele	eaf	overall_breast_cancer
3	rs6762558	4742251	G	A	0.412		0.997	rs6762558:4742251:A:G	rs6762558	A	G	0.380	0.062
3	rs552647	27353716	C	A	0.475		0.999	rs552647:27353716:C:A	rs552647	C	A	0.526	0.075
3	rs62255657	27388664	G	C	0.253		0.994	rs62255657:27388664:C:G	rs62255657	C	G	0.274	0.050
3	rs112476261	29294845	T	C	0.015		0.972	rs112476261:29294845:C:T	rs112476261	C	T	0.016	-0.128
3	rs17838698	30684907	T	C	0.285	106		rs17838698:30684907:C:T	rs17838698	C	T	0.298	0.059
3	rs56387622	46888198	C	T	0.097		0.988	rs56387622:46888198:T:C	rs56387622	T	C	0.103	-0.081
3	rs371314787	49709912	CT	C	0.269		0.957	3:49709912:C:CT	rs371314787	C	CT	0.287	-0.037
3	rs138866686	55970777	AT	A	0.024		0.966	rs138866686:55970777:A:AT	rs138866686	A	AT	0.031	-0.120
3	rs2886671	59373745	T	C	0.421		0.996	rs2886671:59373745:C:T	rs2886671	C	T	0.429	-0.039
3	rs9825432	71620370	T	G	0.360		0.996	rs9825432:71620370:T:G	rs9825432	T	G	0.638	-0.037
3	rs13066793	87037543	G	A	0.090	106		rs13066793:87037543:A:G	rs13066793	A	G	0.092	-0.072
3	rs639355	99403877	A	G	0.480		0.996	rs639355:99403877:G:A	rs639355	G	A	0.485	-0.038
3	3:141112859_CTT_C	141112859	C	CTT	0.431		0.997	rs34207738:141112859:CTT:C	rs34207738	CTT	C	0.415	0.055
3	rs58058861	172285237	A	G	0.220		0.990	rs58058861:172285237:G:A	rs58058861	G	A	0.213	0.042
3	rs9882792	189774456	T	C	0.221		0.985	rs9882792:189774456:C:T	rs9882792	C	T	0.224	-0.048
4	rs10012017	38784633	T	G	0.200		0.996	rs10012017:38784633:G:T	rs10012017	G	T	0.249	0.049
4	rs17014016	89240476	A	G	0.443	106		rs17014016:89240476:G:A	rs17014016	G	A	0.440	0.035
4	rs775780062	92594859	T	TTCTTTC	0.443		0.950	rs147404208:92594859:TTCTTTC:T	rs147404208	TTCTTTC	T	0.445	-0.041
4	rs62331150	106069013	T	G	0.203		0.998	rs62331150:106069013:G:T	rs62331150	G	T	0.229	0.047
4	rs56039025	143467195	T	C	0.113		0.999	rs56039025:143467195:C:T	rs56039025	C	T	0.112	-0.057
4	rs745707748	151218296	CATATTT	C	0.360		0.994	rs138786872:151218296:CATATTT:C	rs138786872	CATATTT	C	0.653	0.039
4	rs28436676	175842495	A	G	0.119		0.998	rs28436676:175842495:G:A	rs28436676	G	A	0.116	-0.090
4	rs62334414	175847436	A	C	0.340		0.987	rs62334414:175847436:C:A	rs62334414	C	A	0.343	0.035
5	rs62641919	345109	C	T	0.059		0.988	rs116095464:345109:T:C	rs116095464	T	C	0.054	0.084
5	rs10069690	1279790	T	C	0.257	106		rs10069690:1279790:C:T	rs10069690	C	T	0.259	0.062
5	rs3215401	1296255	AG	A	0.314		0.996	rs3215401:1296255:A:AG	rs3215401	A	AG	0.307	-0.055

© 2020 Al Ajmi K et al. *JAMA Network Open.*

eTable 2. 305 SNPs used in calculating the polygenic risk scores of the UK biobank females													
CHR	rs_id	BP	A1	A2	MAF	QC-test	Info_score	phase3_1kg_id	rs_number	reference_allele	effect_allele	eaf	overall_breast_cancer
5	rs62329727	1353077	C	T	0.011		0.934	rs62329727:1353077:T:C	rs62329727	T	C	0.012	0.155
5	rs4866496	2777029	A	G	0.422		0.996	rs4866496:2777029:G:A	rs4866496	G	A	0.414	0.039
5	rs17611291	16231194	G	C	0.449		0.994	rs17611291:16231194:G:C	rs17611291	G	C	0.559	-0.043
5	rs770436441	32579616	T	TCA	0.474		0.994	rs35130031:32579616:TCA:T	rs35130031	TCA	T	0.484	0.036
5	rs138335056	44508264	GT	G	0.111		0.987	rs58166936:44508264:G:GT	rs58166936	G	GT	0.127	-0.118
5	rs187108781	44619502	G	A	0.154		0.978	rs187108781:44619502:A:G	rs187108781	A	G	0.155	-0.110
5	rs4613718	44649944	C	T	0.392		0.983	rs4613718:44649944:C:T	rs4613718	C	T	0.601	0.049
5	rs10941679	44706498	G	A	0.252		0.979	rs10941679:44706498:A:G	rs10941679	A	G	0.248	0.050
5	rs17343002	44853593	C	G	0.314	106		rs17343002:44853593:G:C	rs17343002	G	C	0.308	-0.034
5	rs553874618	55662540	CT	C	0.355		0.974	rs113803968:55662540:C:CT	rs113803968	C	CT	0.363	-0.046
5	rs889310	55965167	C	T	0.438		0.988	rs889310:55965167:C:T	rs889310	C	T	0.558	0.039
5	rs16886165	56023083	G	T	0.163	106		rs16886165:56023083:T:G	rs16886165	T	G	0.158	0.137
5	rs76250845	56042972	T	C	0.059		0.995	rs76250845:56042972:C:T	rs76250845	C	T	0.052	0.087
5	rs11949391	56045081	C	T	0.165		0.992	rs11949391:56045081:T:C	rs11949391	T	C	0.166	-0.056
5	rs113778879	58241712	C	T	0.429		0.944	rs113778879:58241712:C:T	rs113778879	C	T	0.575	-0.043
5	rs3010266	71965007	A	G	0.257		0.975	rs3010266:71965007:G:A	rs3010266	G	A	0.257	-0.041
5	rs157557	73234583	C	T	0.323		0.956	rs157557:73234583:T:C	rs157557	T	C	0.321	-0.036
5	rs767431357	77155397	G	GT	0.344		0.983	rs144028731:77155397:GT:G	rs144028731	GT	G	0.347	-0.041
5	rs34525310	79180995	GA	G	0.177		0.997	rs34525310:79180995:G:GA	rs34525310	G	GA	0.176	0.033
5	5:81512947_TA_T	81512947	T	TA	0.249		0.999	rs146817970:81512947:TA:T	rs146817970	TA	T	0.250	-0.060
5	rs332529	90789470	A	G	0.148		0.986	rs332529:90789470:G:A	rs332529	G	A	0.158	-0.056
5	rs17157372	104300273	T	G	0.167		0.987	rs17157372:104300273:G:T	rs17157372	G	T	0.181	-0.049
5	rs335160	122478676	C	A	0.250		0.998	rs335160:122478676:C:A	rs335160	C	A	0.745	-0.039
5	rs1428387	122705244	T	C	0.023		0.997	rs1428387:122705244:C:T	rs1428387	C	T	0.031	0.094
5	rs6860806	131640536	A	G	0.451		0.990	rs6860806:131640536:A:G	rs6860806	A	G	0.543	0.039
5	rs6596100	132407058	T	C	0.237		0.997	rs6596100:132407058:C:T	rs6596100	C	T	0.245	-0.039
5	rs1432679	158244083	C	T	0.448	106		rs1432679:158244083:C:T	rs1432679	C	T	0.568	-0.068

© 2020 Al Ajmi K et al. *JAMA Network Open.*

eTable 2. 305 SNPs used in calculating the polygenic risk scores of the UK biobank females													
CHR	rs_id	BP	A1	A2	MAF	QC-test	Info_score	phase3_1kg_id	rs_number	reference_allele	effect_allele	eaf	overall_breast_cancer
5	rs10074269	169591460	C	T	0.342		0.998	rs10074269:169591460:T:C	rs10074269	T	C	0.335	0.041
5	rs6864691	173358154	A	G	0.422		0.995	rs6864691:173358154:G:A	rs6864691	G	A	0.407	0.037
5	rs4868701	176134882	T	C	0.458		0.988	rs4868701:176134882:T:C	rs4868701	T	C	0.542	0.036
6	rs418053	13713366	G	C	0.425		0.995	rs418053:13713366:G:C	rs418053	G	C	0.569	-0.055
6	rs3819405	16399557	T	C	0.331	106		rs3819405:16399557:C:T	rs3819405	C	T	0.330	-0.037
6	rs12211970	18783140	G	A	0.390		0.998	rs12211970:18783140:G:A	rs12211970	G	A	0.620	0.033
6	rs769485514	20537845	C	CA	0.497		0.895	6:20537845:CA:C	rs543824204	CA	C	0.473	-0.039
6	rs9358466	21923810	C	T	0.433		0.996	rs9358466:21923810:T:C	rs9358466	T	C	0.430	-0.032
6	rs34196306	27425644	C	G	0.111		0.980	rs34196306:27425644:G:C	rs34196306	G	C	0.082	-0.074
6	rs111342015	43227141	A	G	0.100	106		rs111342015:43227141:G:A	rs111342015	G	A	0.099	-0.064
6	rs10623112	82263549	A	AAT	0.410		0.946	6:82263549:AAT:A	rs796893180	AAT	A	0.426	0.048
6	6:85912194_CAA_C	85912194	C	CAA	0.063		0.973	rs146519950:85912194:CAA:C	rs146519950	CAA	C	0.060	0.076
6	rs73754909	87803819	C	T	0.274		0.987	rs73754909:87803819:T:C	rs73754909	T	C	0.277	0.038
6	rs55941023	130341728	C	CT	0.299		0.985	rs55941023:130341728:C:CT	rs55941023	C	CT	0.712	0.047
6	rs2121348	149595505	C	T	0.199		0.996	rs2121348:149595505:T:C	rs2121348	T	C	0.206	-0.048
6	rs6913578	151949806	C	A	0.323		0.998	rs6913578:151949806:A:C	rs6913578	A	C	0.308	0.070
6	rs60954078	151955914	G	A	0.071		0.997	rs60954078:151955914:A:G	rs60954078	A	G	0.071	0.145
6	6:152022664_CAAAAAAA_C	152022664	CAAAAAAA	C	0.375		0.919	rs57589542:152022664:CAAAAAAA:C	rs57589542	CAAAAAAA	C	0.612	0.014
6	rs851984	152023191	A	G	0.391		0.989	rs851984:152023191:G:A	rs851984	G	A	0.397	0.063
6	rs6904031	152055978	T	A	0.056		0.984	rs6904031:152055978:A:T	rs6904031	A	T	0.063	0.074
6	rs910416	152432902	C	T	0.489		0.991	rs910416:152432902:C:T	rs910416	C	T	0.515	0.065
6	rs9364472	169006947	C	G	0.493		0.982	rs9364472:169006947:C:G	rs9364472	C	G	0.520	-0.031
6	rs6940159	170332621	T	C	0.368		0.945	rs6940159:170332621:T:C	rs6940159	T	C	0.616	0.037
7	rs7971	21940960	G	A	0.358		0.993	rs7971:21940960:A:G	rs7971	A	G	0.352	-0.047
7	rs289997	25569548	T	C	0.145		0.993	rs289997:25569548:C:T	rs289997	C	T	0.167	-0.049
7	rs74765302	28869017	A	G	0.108		0.991	rs74765302:28869017:G:A	rs74765302	G	A	0.107	-0.057
7	rs13244925	55192256	A	C	0.432		0.995	rs13244925:55192256:A:C	rs13244925	A	C	0.550	-0.035
7	rs17268829	94113799	C	T	0.298		0.998	rs17268829:94113799:T:C	rs17268829	T	C	0.279	0.045

© 2020 Al Ajmi K et al. *JAMA Network Open.*

eTable 2. 305 SNPs used in calculating the polygenic risk scores of the UK biobank females													
CHR	rs_id	BP	A1	A2	MAF	QC-test	Info_score	phase3_1kg_id	rs_number	reference_allele	effect_allele	eaf	overall_breast_cancer
7	rs4439053	98005235	A	G	0.169		0.982	rs4439053:98005235:G:A	rs4439053	G	A	0.163	-0.047
7	rs111963714	99948655	G	T	0.209		0.989	rs111963714:99948655:T:G	rs111963714	T	G	0.211	0.042
7	rs71559437	101552440	A	G	0.132		0.976	rs71559437:101552440:G:A	rs71559437	G	A	0.126	-0.057
7	rs7800548	102481842	C	T	0.338		0.999	rs7800548:102481842:T:C	rs7800548	T	C	0.342	0.042
7	rs12706954	130656911	T	C	0.382		0.950	rs12706954:130656911:C:T	rs12706954	C	T	0.373	-0.048
7	rs68056147	130674481	A	G	0.301		0.969	rs68056147:130674481:G:A	rs68056147	G	A	0.297	0.042
7	rs201664599	139943702	CT	C	0.453		0.950	rs5887960:139943702:CT:C	rs5887960	CT	C	0.538	0.058
7	rs62485509	144048902	T	G	0.200		0.887	rs62485509:144048902:G:T	rs62485509	G	T	0.228	-0.056
8	rs66823261	170692	C	T	0.216		0.969	rs66823261:170692:T:C	rs66823261	T	C	0.223	0.048
8	8:17787610_CT_C	17787610	CT	C	0.360		0.949	rs3988353:17787610:CT:C	rs3988353	CT	C	0.623	-0.038
8	rs1028016	23447496	A	G	0.375		0.998	rs1028016:23447496:A:G	rs1028016	A	G	0.649	-0.039
8	rs310295	23663653	A	C	0.421		0.994	rs310295:23663653:C:A	rs310295	C	A	0.403	0.034
8	rs9693444	29509616	A	C	0.325	106		rs9693444:29509616:A:C	rs9693444	A	C	0.676	-0.060
8	rs13365225	36858483	G	A	0.160		0.997	rs13365225:36858483:A:G	rs13365225	A	G	0.182	-0.076
8	rs1511243	76230943	A	G	0.177		0.999	rs1511243:76230943:A:G	rs1511243	A	G	0.828	0.076
8	rs72658084	76333056	T	C	0.091		0.991	rs72658084:76333056:C:T	rs72658084	C	T	0.088	0.113
8	rs1533366	76378165	T	G	0.361		0.996	rs1533366:76378165:G:T	rs1533366	G	T	0.360	-0.039
8	rs62517052	102483100	C	T	0.095		0.979	rs62517052:102483100:T:C	rs62517052	T	C	0.097	0.059
8	rs12546444	106358620	T	A	0.093		0.966	rs12546444:106358620:A:T	rs12546444	A	T	0.100	-0.075
8	rs13267382	117209548	A	G	0.345		0.985	rs13267382:117209548:A:G	rs13267382	A	G	0.645	-0.042
8	rs62526620	120862186	G	A	0.129		0.988	rs62526620:120862186:A:G	rs62526620	A	G	0.132	0.053
8	rs35542655	124563705	C	T	0.144		0.978	rs35542655:124563705:T:C	rs35542655	T	C	0.146	0.048
8	rs12541094	124571581	A	G	0.415	106		rs12541094:124571581:G:A	rs12541094	G	A	0.417	0.034
8	rs7842619	124739913	G	T	0.414		0.997	rs7842619:124739913:T:G	rs7842619	T	G	0.399	0.047
8	rs35961416	128213561	CA	C	0.402		0.883	rs35961416:128213561:C:CA	rs35961416	C	CA	0.415	-0.043
8	rs12550713	128370949	G	C	0.401		0.999	rs12550713:128370949:C:G	rs12550713	C	G	0.402	0.064
8	rs10096351	128372172	A	G	0.457		0.999	rs10096351:128372172:A:G	rs10096351	A	G	0.545	0.060
8	rs1016578	129199566	A	G	0.177		0.997	rs1016578:129199566:G:A	rs1016578	G	A	0.172	0.062
8	rs7830152	143669254	G	A	0.336		0.984	rs7830152:143669254:A:G	rs7830152	A	G	0.339	-0.035
9	rs10975870	6880263	G	A	0.291		0.991	rs10975870:6880263:A:G	rs10975870	A	G	0.286	0.035

© 2020 Al Ajmi K et al. *JAMA Network Open.*

eTable 2. 305 SNPs used in calculating the polygenic risk scores of the UK biobank females													
CHR	rs_id	BP	A1	A2	MAF	QC-test	Info_score	phase3_1kg_id	rs_number	reference_allele	effect_allele	eaf	overall_breast_cancer
9	rs745322029	21964882	C	CAAAA	0.333		0.966	rs3057314:21964882:CAAAA:C	rs3057314	CAAAA	C	0.318	0.055
9	rs17694493	22041998	G	C	0.133		0.982	rs17694493:22041998:C:G	rs17694493	C	G	0.139	0.029
9	rs4880038	36928288	T	C	0.443		1.000	rs4880038:36928288:T:C	rs4880038	T	C	0.535	0.025
9	rs665889	87782211	T	C	0.493		0.994	rs665889:87782211:T:C	rs665889	T	C	0.509	0.036
9	rs10120432	98362587	C	T	0.092		0.991	rs10120432:98362587:T:C	rs10120432	T	C	0.094	0.058
9	9:110303808_TAA_T	110303808	T	TAA	0.180		0.983	rs60037937:110303808:TAA:T	rs60037937	TAA	T	0.207	0.080
9	rs10816625	110837073	G	A	0.063	106		rs10816625:110837073:A:G	rs10816625	A	G	0.063	0.116
9	rs13294895	110837176	T	C	0.169		0.942	rs13294895:110837176:C:T	rs13294895	C	T	0.175	0.065
9	rs7848334	110849525	G	T	0.393		0.935	rs7848334:110849525:G:T	rs7848334	G	T	0.598	0.015
9	rs630965	110885479	C	T	0.384		0.996	rs630965:110885479:C:T	rs630965	C	T	0.622	0.088
9	rs1895062	119313486	G	A	0.407		0.993	rs1895062:119313486:A:G	rs1895062	A	G	0.409	-0.046
9	rs3861871	129424719	G	A	0.452		0.976	rs3861871:129424719:A:G	rs3861871	A	G	0.458	-0.038
9	rs550057	136146597	T	C	0.256		0.999	9:136146597:C:T	rs550057	C	T	0.273	0.040
10	rs55910451	5794652	G	A	0.223		0.996	rs55910451:5794652:A:G	rs55910451	A	G	0.214	0.047
10	rs10796139	13892298	A	G	0.448		0.998	rs10796139:13892298:G:A	rs10796139	G	A	0.438	0.037
10	rs7072776	22032942	A	G	0.275	106		rs7072776:22032942:A:G	rs7072776	A	G	0.709	-0.058
10	rs762131501	22477776	A	ACC	0.016		0.980	10:22477776:ACC:A	rs542275778	ACC	A	0.020	0.169
10	rs2384736	38523626	A	C	0.375		0.966	rs2384736:38523626:C:A	rs2384736	C	A	0.370	0.040
10	rs10995201	64299890	G	A	0.142		0.977	rs10995201:64299890:A:G	rs10995201	A	G	0.160	-0.135
10	rs6479868	64819996	T	G	0.195		0.989	rs6479868:64819996:G:T	rs6479868	G	T	0.196	0.047
10	rs111833376	71335574	T	C	0.316		0.958	rs111833376:71335574:C:T	rs111833376	C	T	0.318	-0.040
10	rs719338	80851257	G	T	0.405		0.991	rs719338:80851257:G:T	rs719338	G	T	0.617	-0.081
10	rs4980029	80886726	G	A	0.157		0.988	rs4980029:80886726:A:G	rs4980029	A	G	0.163	0.076
10	10:95292187_CAA_C	95292187	CAA	C	0.181		0.964	rs140936696:95292187:CAA:C	rs140936696	CAA	C	0.823	-0.051
10	rs10885405	114777670	T	C	0.465		0.999	rs10885405:114777670:C:T	rs10885405	C	T	0.463	0.047
10	rs12250948	115128491	T	C	0.208		0.984	rs12250948:115128491:T:C	rs12250948	T	C	0.785	-0.059
10	rs9421410	123095209	A	G	0.314		0.988	rs9421410:123095209:G:A	rs9421410	G	A	0.327	-0.054
10	rs45631580	123340107	G	A	0.058		0.993	rs45631580:123340107:A:G	rs45631580	A	G	0.066	0.151

© 2020 Al Ajmi K et al. *JAMA Network Open.*

eTable 2. 305 SNPs used in calculating the polygenic risk scores of the UK biobank females													
CHR	rs_id	BP	A1	A2	MAF	QC-test	Info_score	phase3_1kg_id	rs_number	reference_allele	effect_allele	eaf	overall_breast_cancer
10	10:123340431_GC_G	123340431	GC	G	0.404		0.993	rs35054928:123340431:GC:G	rs35054928	GC	G	0.596	-0.241
10	rs45631563	123349324	T	A	0.043		0.975	rs45631563:123349324:A:T	rs45631563	A	T	0.048	-0.261
11	rs7394715	433617	T	C	0.207		0.993	rs7394715:433617:T:C	rs7394715	T	C	0.797	-0.044
11	rs6597981	803017	A	G	0.478	106		rs6597981:803017:A:G	rs6597981	A	G	0.517	0.046
11	rs4980386	1895708	A	C	0.378		0.995	rs4980386:1895708:C:A	rs4980386	C	A	0.392	-0.076
11	rs10832963	18664241	T	G	0.253		0.995	rs10832963:18664241:T:G	rs10832963	T	G	0.729	0.046
11	rs4472923	42844441	T	C	0.330		0.998	rs4472923:42844441:C:T	rs4472923	C	T	0.328	-0.034
11	rs10838267	44368892	G	A	0.455		0.992	rs10838267:44368892:G:A	rs10838267	G	A	0.550	0.037
11	rs77047825	46318032	G	C	0.071		0.976	rs77047825:46318032:C:G	rs77047825	C	G	0.066	-0.075
11	rs12287832	65553492	A	C	0.192		0.995	rs12287832:65553492:C:A	rs12287832	C	A	0.187	0.043
11	rs10896047	65572431	A	G	0.472		0.988	rs10896047:65572431:G:A	rs10896047	G	A	0.489	-0.035
11	rs35039974	69328130	T	A	0.200		0.981	rs35039974:69328130:A:T	rs35039974	A	T	0.213	-0.042
11	rs661204	69330983	A	G	0.119		0.991	rs661204:69330983:G:A	rs661204	G	A	0.125	0.102
11	rs78540526	69331418	T	C	0.071		0.988	rs78540526:69331418:C:T	rs78540526	C	T	0.075	0.178
11	rs7125780	103614438	T	G	0.356		0.992	rs7125780:103614438:T:G	rs7125780	T	G	0.657	0.015
11	rs199504893	108267402	CA	C	0.408		0.999	rs199504893:108267402:C:CA	rs199504893	C	CA	0.417	-0.002
11	rs610437	111696440	T	C	0.379		0.999	rs610437:111696440:T:C	rs610437	T	C	0.622	-0.040
11	rs625145	116727936	T	A	0.188		0.999	rs625145:116727936:A:T	rs625145	A	T	0.205	-0.042
11	rs7121616	122966626	G	A	0.281		0.997	rs7121616:122966626:A:G	rs7121616	A	G	0.292	-0.038
11	rs7939702	129243417	T	G	0.142		0.985	rs7939702:129243417:T:G	rs7939702	T	G	0.862	-0.054
11	rs11822830	129461016	A	G	0.415	106		rs11822830:129461016:A:G	rs11822830	A	G	0.602	0.045
12	rs797736	293626	G	A	0.368		0.995	12:293626:A:G	rs797736	A	G	0.371	0.040
12	rs12422552	14413931	C	G	0.266	106		12:14413931:G:C	rs12422552	G	C	0.262	0.048
12	rs788458	28149568	T	C	0.124		0.997	12:28149568:C:T	rs788458	C	T	0.117	-0.062
12	rs7297051	28174817	T	C	0.228		0.969	12:28174817:C:T	rs7297051	C	T	0.242	-0.086
12	rs11049431	28347382	T	C	0.222		0.976	12:28347382:C:T	rs11049431	C	T	0.215	-0.052
12	rs1027113	29140260	G	A	0.076		0.988	12:29140260:G:A	rs1027113	G	A	0.913	0.065
12	rs2277339	57146069	G	T	0.103	106		12:57146069:T:G	rs2277339	T	G	0.104	-0.058
12	rs2870876	70798355	T	A	0.187		0.996	12:70798355:A:T	rs2870876	A	T	0.181	0.047
12	rs111622698	83064195	GA	G	0.102		0.966	12:83064195:G:GA	rs111622698	G	GA	0.099	0.067

© 2020 Al Ajmi K et al. *JAMA Network Open.*

eTable 2. 305 SNPs used in calculating the polygenic risk scores of the UK biobank females													
CHR	rs_id	BP	A1	A2	MAF	QC-test	Info_score	phase3_1kg_id	rs_number	reference_allele	effect_allele	eaf	overall_breast_cancer
12	rs10862899	85004551	C	T	0.490		0.997	12:85004551:C:T	rs10862899	C	T	0.496	0.035
12	rs17356907	96027759	G	A	0.304	106		12:96027759:A:G	rs17356907	A	G	0.296	-0.087
12	rs7132703	103097887	T	C	0.114		0.987	12:103097887:C:T	rs7132703	C	T	0.118	0.055
12	rs11065822	111600134	T	G	0.348		0.948	12:111600134:G:T	rs11065822	G	T	0.372	-0.044
12	rs1061657	115108136	C	T	0.257	106		12:115108136:T:C	rs1061657	T	C	0.262	0.047
12	rs11067551	115796577	G	A	0.194	106		12:115796577:A:G	rs11067551	A	G	0.196	-0.043
12	rs2454399	115835836	C	T	0.422		0.993	12:115835836:T:C	rs2454399	T	C	0.417	-0.081
12	rs206966	120832146	T	C	0.143		0.927	12:120832146:C:T	rs206966	C	T	0.159	0.052
13	rs56404467	32839990	A	G	0.016		0.903	rs56404467:32839990:G:A	rs56404467	G	A	0.017	0.042
13	rs11571833	32972626	T	A	0.009	106		rs11571833:32972626:A:T	rs11571833	A	T	0.008	0.269
13	rs9315973	43501356	A	G	0.168		0.982	rs9315973:43501356:A:G	rs9315973	A	G	0.830	0.052
13	rs12870942	73806982	C	T	0.322		0.994	rs12870942:73806982:T:C	rs12870942	T	C	0.315	0.035
13	rs2181965	73960952	A	G	0.241		0.998	rs2181965:73960952:A:G	rs2181965	A	G	0.762	0.040
14	rs34914085	37128564	A	C	0.210		0.993	rs34914085:37128564:C:A	rs34914085	C	A	0.212	-0.073
14	rs2253012	37228504	T	C	0.440		0.990	rs2253012:37228504:C:T	rs2253012	C	T	0.443	0.039
14	rs2588809	68660428	T	C	0.156	106		rs2588809:68660428:T:C	rs2588809	T	C	0.835	-0.047
14	rs11624333	68979835	C	T	0.282		0.980	rs11624333:68979835:T:C	rs11624333	T	C	0.258	-0.091
14	14:91751788_TC_T	91751788	TC	T	0.313		0.983	rs11341843:91751788:TC:T	rs11341843	TC	T	0.693	0.038
14	rs941764	91841069	G	A	0.342	106		rs941764:91841069:A:G	rs941764	A	G	0.344	0.051
14	rs78440108	93070286	T	C	0.175		0.974	rs78440108:93070286:C:T	rs78440108	C	T	0.171	-0.058
14	rs4983544	105213978	G	T	0.446		0.992	rs4983544:105213978:T:G	rs4983544	T	G	0.459	0.040
15	rs187010898	46680811	A	C	0.012		0.966	rs187010898:46680811:C:A	rs187010898	C	A	0.012	-0.197
15	rs4774565	50694306	G	A	0.326		0.974	rs4774565:50694306:A:G	rs4774565	A	G	0.345	-0.042
15	rs8042593	66630569	G	A	0.351		0.999	rs8042593:66630569:G:A	rs8042593	G	A	0.641	-0.037
15	rs35874463	67457698	G	A	0.058	106		rs35874463:67457698:A:G	rs35874463	A	G	0.050	0.078
15	rs8035987	75750383	C	T	0.250		0.985	rs8035987:75750383:T:C	rs8035987	T	C	0.260	-0.041
15	rs2290202	91512267	T	G	0.128		0.994	rs2290202:91512267:G:T	rs2290202	G	T	0.135	-0.059
15	rs144767203	100905819	C	A	0.107		0.992	rs144767203:100905819:A:C	rs144767203	A	C	0.110	-0.061
16	16:4008542_CAAAAA_C	4008542	CAAAAA	C	0.168		0.950	rs57920543:4008542:CAAAAA:C	rs57920543	CAAAAA	C	0.821	-0.033
16	rs11076805	4106788	A	C	0.262		0.971	rs11076805:4106788:C:A	rs11076805	C	A	0.264	-0.030

© 2020 Al Ajmi K et al. *JAMA Network Open.*

eTable 2. 305 SNPs used in calculating the polygenic risk scores of the UK biobank females													
CHR	rs_id	BP	A1	A2	MAF	QC-test	Info_score	phase3_1kg_id	rs_number	reference_allele	effect_allele	eaf	overall_breast_cancer
16	rs12709163	6963972	C	G	0.188		0.964	rs12709163:6963972:C:G	rs12709163	C	G	0.784	0.035
16	rs34872983	10706580	A	G	0.061		0.984	rs34872983:10706580:G:A	rs34872983	G	A	0.070	-0.074
16	rs75753503	23007047	T	G	0.022		0.982	rs75753503:23007047:G:T	rs75753503	G	T	0.024	0.122
16	rs35668161	52538825	A	C	0.249		0.992	rs35668161:52538825:C:A	rs35668161	C	A	0.256	0.115
16	rs4784227	52599188	T	C	0.239	106		rs4784227:52599188:C:T	rs4784227	C	T	0.241	0.107
16	rs55872725	53809123	T	C	0.401		1.000	rs55872725:53809123:C:T	rs55872725	C	T	0.420	-0.070
16	rs6499648	53861139	C	T	0.227		0.997	rs6499648:53861139:C:T	rs6499648	C	T	0.760	-0.034
16	rs7184573	53861592	A	G	0.373		0.979	rs7184573:53861592:G:A	rs7184573	G	A	0.366	-0.034
16	rs28539243	54682064	A	G	0.488	106		rs28539243:54682064:G:A	rs28539243	G	A	0.485	0.048
16	rs7500067	80648296	G	A	0.222		0.994	rs7500067:80648296:A:G	rs7500067	A	G	0.230	0.084
16	rs9931038	85145977	C	T	0.487	106		rs9931038:85145977:T:C	rs9931038	T	C	0.486	-0.021
16	rs12449271	87086492	C	T	0.253		0.989	rs12449271:87086492:T:C	rs12449271	T	C	0.259	-0.047
17	rs79461387	29168077	T	G	0.260		0.986	rs79461387:29168077:G:T	rs79461387	G	T	0.261	-0.057
17	rs150537328	39251123	C	T	0.062		0.955	rs150537328:39251123:T:C	rs150537328	T	C	0.068	0.080
17	rs11296	40127060	C	T	0.051		0.942	rs11296:40127060:T:C	rs11296	T	C	0.057	0.017
17	rs17881320	40485239	T	G	0.089		0.987	rs17881320:40485239:G:T	rs17881320	G	T	0.087	-0.057
17	rs149370081	40744470	A	G	0.015		0.987	rs149370081:40744470:G:A	rs149370081	G	A	0.012	0.202
17	rs545502941	43212339	CT	C	0.211		0.973	rs71363517:43212339:C:CT	rs71363517	C	CT	0.228	0.044
17	rs2668667	44283858	A	G	0.132		0.864	17:44283858:G:A	rs2668667	G	A	0.190	-0.054
17	rs2787486	53209774	C	A	0.314		0.991	rs2787486:53209774:A:C	rs2787486	A	C	0.302	-0.079
17	rs745570	77781725	A	G	0.485		0.999	rs745570:77781725:A:G	rs745570	A	G	0.504	-0.040
18	rs16976596	11696613	T	C	0.126		0.968	rs16976596:11696613:C:T	rs16976596	C	T	0.138	-0.038
18	rs11665269	20634253	C	T	0.368		0.980	rs11665269:20634253:C:T	rs11665269	C	T	0.640	-0.042
18	rs1111207	24125857	C	T	0.431		0.996	rs1111207:24125857:T:C	rs1111207	T	C	0.421	0.035
18	rs527616	24337424	C	G	0.360	106		rs527616:24337424:C:G	rs527616	C	G	0.621	0.046
18	18:24518050_AT_A	24518050	A	AT	0.287		0.989	rs35369219:24518050:AT:A	rs35369219	AT	A	0.277	-0.060
18	rs8092192	25407513	C	G	0.295		0.992	rs8092192:25407513:C:G	rs8092192	C	G	0.713	0.040
18	rs72931898	29981526	A	G	0.044		0.993	rs72931898:29981526:G:A	rs72931898	G	A	0.047	-0.106
18	rs9954058	42411803	C	G	0.079		0.995	rs9954058:42411803:G:C	rs9954058	G	C	0.072	-0.088
18	rs9952980	42888797	C	T	0.356		0.976	rs9952980:42888797:T:C	rs9952980	T	C	0.352	-0.054
19	rs117922601	13249921	T	G	0.056		0.993	rs117922601:13249921:G:T	rs117922601	G	T	0.051	0.096

© 2020 Al Ajmi K et al. *JAMA Network Open.*

eTable 2. 305 SNPs used in calculating the polygenic risk scores of the UK biobank females													
CHR	rs_id	BP	A1	A2	MAF	QC-test	Info_score	phase3_1kg_id	rs_number	reference_allele	effect_allele	eaf	overall_breast_cancer
19	rs56069439	17393925	A	C	0.295		1.000	rs56069439:17393925:C:A	rs56069439	C	A	0.296	0.038
19	rs10164323	18569492	T	C	0.354		0.999	rs10164323:18569492:C:T	rs10164323	C	T	0.348	-0.072
19	rs140702307	19517054	CGGGCG	C	0.355		0.998	rs140702307:19517054:C:CGGGCG	rs140702307	C	CGGGCG	0.354	0.044
19	rs56681946	44283031	C	T	0.378		0.998	rs56681946:44283031:T:C	rs56681946	T	C	0.352	0.062
19	rs4399645	46166073	T	C	0.409		0.983	rs4399645:46166073:T:C	rs4399645	T	C	0.607	-0.036
19	rs1172821	55816678	T	C	0.372	106		rs1172821:55816678:C:T	rs1172821	C	T	0.363	-0.036
20	rs16991615	5948227	A	G	0.065	106		rs16991615:5948227:G:A	rs16991615	G	A	0.063	0.076
20	rs1154723	11379842	T	C	0.051	106		rs1154723:11379842:T:C	rs1154723	T	C	0.948	0.084
20	rs6030585	41613706	C	G	0.202		0.979	rs6030585:41613706:C:G	rs6030585	C	G	0.793	0.032
20	rs13039563	52296849	A	G	0.240		0.987	rs13039563:52296849:G:A	rs13039563	G	A	0.240	0.044
21	rs2822999	16364756	G	T	0.180		0.991	rs2822999:16364756:T:G	rs2822999	T	G	0.173	0.065
21	rs2823130	16566350	G	A	0.087	106		rs2823130:16566350:A:G	rs2823130	A	G	0.087	0.060
21	rs2403907	16574455	A	C	0.314		0.992	rs2403907:16574455:C:A	rs2403907	C	A	0.317	-0.071
21	rs4818836	47762932	A	G	0.031		0.976	rs4818836:47762932:G:A	rs4818836	G	A	0.036	0.095
22	rs9798754	19766137	T	C	0.365		0.986	rs9798754:19766137:C:T	rs9798754	C	T	0.380	-0.037
22	rs17879961	29121087	G	A	0.000	106		rs17879961:29121087:A:G	rs17879961	A	G	0.005	0.184
22	rs5997390	29135543	A	G	0.080		0.992	rs5997390:29135543:G:A	rs5997390	G	A	0.087	0.065
22	rs34134147	29203724	T	C	0.021		0.981	rs34134147:29203724:C:T	rs34134147	C	T	0.021	0.141
22	rs132289	29551872	A	G	0.020		0.948	rs132289:29551872:A:G	rs132289	A	G	0.985	-0.172
22	rs5750715	39343916	A	T	0.251		0.990	rs5750715:39343916:T:A	rs5750715	T	A	0.254	0.041
22	22:40904707_CT_C	40904707	C	CT	0.093		0.984	rs66987842:40904707:CT:C	rs66987842	CT	C	0.110	0.115
22	rs9611990	43433100	T	C	0.121		0.990	rs9611990:43433100:C:T	rs9611990	C	T	0.114	-0.060
22	rs112855987	45319953	A	G	0.420		0.983	rs112855987:45319953:G:A	rs112855987	G	A	0.417	-0.013
22	rs28512361	46283297	A	G	0.105		0.985	rs28512361:46283297:G:A	rs28512361	G	A	0.112	0.074

rs_id	Genetic variant identifier
BP	Build 37 position
A1	Minor allele
MAF	Minor allele frequency
QC-test	Result of marker-based QC test by UKBiobank (See Marker-based QC, 2.1.4 in QC report) for directly genotyped variants

© 2020 Al Ajmi K et al. *JAMA Network Open.*

Info_score	Information score for imputed variants
phase3_1kg_id	Identifier from Phase 3 of 1000 Genomes
Variables taken from Mavaddat *et al.* (2019) study [38]:	
reference_allele	
effect_allele	
eaf	Effect Allele frequency estimates for controls in the complete Oncoarray dataset
overall_breast_cancer	Coefficients for best model for overall breast cancer

© 2020 Al Ajmi K et al. *JAMA Network Open.*
